# Novel online daily diary interventions for nonsuicidal self-injury: a randomized controlled trial

**DOI:** 10.1186/s12888-018-1840-6

**Published:** 2018-08-22

**Authors:** Jill M. Hooley, Kathryn R. Fox, Shirley B. Wang, Anita N. D. Kwashie

**Affiliations:** Harvard University, Cambridge, MA USA

**Keywords:** Self-criticism, Expressive writing, Depression, Suicide

## Abstract

**Background:**

Nonsuicidal self-injury (NSSI), which involves self-damaging behavior (e.g., cutting) causes tissue damage and places people at elevated risk for future suicidal behaviors. Yet few specific treatments for NSSI currently exist. Extreme self-criticism is implicated in the development and maintenance of NSSI. We conducted a randomized controlled trial to evaluate Autobiographical Self-Enhancement Training (ASET), a novel, cognitive intervention for NSSI focused on reducing self-criticism and enhancing positive self-worth. We also examined whether Expressive Writing (EW) was a helpful treatment for NSSI.

**Method:**

Participants (*N* = 144) who had engaged in NSSI at least twice in the past month were recruited online and then randomly assigned via Qualtrics to receive the ASET intervention (*N* = 49), the EW intervention (*N* = 49), or Daily Journaling [JNL; *N* = 46]), an active comparison condition. Treatments were designed as month-long daily diaries. Participants in ASET wrote about something that made them feel good about themselves that day, participants in EW described something that had been on their mind that day, and participants in JNL reported on the events of the day in a factually descriptive manner without emotional content.

**Results:**

Intent-to-treat analyses revealed that, regardless of treatment group, participants showed significant reductions in self-criticism, NSSI episodes, depression, and suicide ideation from baseline to the end of active treatment. Relative to the JNL group, the ASET group reported significantly less self-criticism at post-treatment; this was not maintained at follow-up. There was also a trend toward ASET being associated with less suicide ideation at the end of treatment compared to EW. This difference was significant at the 3-month follow-up. Unexpectedly, the JNL group reported significantly less suicide ideation than the EW group at post-treatment; this was maintained at 3-month follow-up. No significant treatment effects were detected for suicide plans, suicidal behaviors, desire to discontinue NSSI, or likelihood of future NSSI.

**Conclusion:**

Self-criticism is an important treatment target in NSSI, but changing self-criticism in people with an established history of NSSI presents challenges. Nonetheless, all approaches provided benefits. This study also established the feasibility of inexpensive and easily disseminated treatments for NSSI.

**Trial registration number:**

ISRCTN12276176 (retrospectively registered, March 13, 2018).

## Background

Nonsuicidal self-injury (NSSI) involves intentional and self-directed harm (e.g., self-cutting, burning) that is enacted without suicidal intent [1]. Although it tends to be painful, dangerous, and stigmatized, NSSI is quite common in the general population, with lifetime prevalence rates of approximately 17% in adolescents and 5% in adults [2]. NSSI is associated with both physical and social-emotional harm in the short and long-term. A major concern is the strong link between NSSI and suicidal thoughts and behaviors both concurrently [3, 4] and prospectively [5].

All of this highlights the need for research on NSSI treatments. Unfortunately, few approaches to date have consistently reduced NSSI compared to active control treatments [6–9]. One notable exception is the intervention developed by Franklin and colleagues [10]. This intervention, called Therapeutic Evaluative Conditioning (TEC), uses a form of Pavlovian conditioning [11] to *increase* aversion to self-injury stimuli (e.g., knives, blood) and to *decrease* aversion to the self (i.e., reduce self-criticism). Utilizing an online, app-based treatment and across three randomized control trials, TEC resulted in significant reductions in NSSI, suicide plans, and suicide attempts, compared to an active control treatment.

Franklin and colleagues’ [10] approach is notable for three primary reasons. First, it targeted two relatively novel treatment targets for NSSI based on growing evidence that high self-criticism and low aversion to NSSI stimuli are important NSSI risk factors [12]. Second, TEC reduced both NSSI and suicidal thoughts and behaviors, suggesting that these treatment targets may prove effective for a range of self-injurious thoughts and behaviors. Third, TEC was the first highly scalable, inexpensive, online treatment to be developed for NSSI. Results from Franklin et al.’s [10] study demonstrated that (a) targeting new risk factors such as self-criticism and diminished aversion to NSSI stimuli may be effective for reducing NSSI and (b) that it is possible to conduct online treatments for NSSI.

In the present study we sought to use this information to create a new, online treatment program for NSSI. Specifically, we conducted a randomized controlled trial (RCT) to evaluate Autobiographical Self-Enhancement Training (ASET) -- a novel, cognitive intervention for NSSI focused on reducing self-criticism and enhancing positive self-worth. To help reduce NSSI engagement, self-worth was selected as a primary treatment target for several reasons. First, people who engage in NSSI demonstrate lowered levels of self-worth across several domains, including body image [13], self-dissatisfaction [14], and self-criticism [15, 16]. Second, recent longitudinal research found that implicit and explicit self-criticism predicted continued NSSI engagement over a 4-week follow-up period above and beyond other relevant factors [17], suggesting that self-criticism may be an important NSSI risk factor and potential treatment target. Third, experimental research has demonstrated that pain endurance is elevated among people who engage in NSSI and that reducing self-criticism normalizes this [18]. Finally, as noted above, a recent treatment study designed to reduce self-criticism as well as increase aversion to NSSI stimuli decreased NSSI engagement over the treatment period [10]. Given this compelling body of research implicating self-criticism as an important NSSI risk factor, we hypothesized that decreasing self-criticism would result in lowered rates of NSSI engagement when compared to treatments targeting other factors. However, unlike the approach adopted by Franklin and colleagues [10] which involved conditioning, we used a more explicit method in an effort to target self-criticism more directly and in a manner that involved participants’ awareness, perhaps therefore improving treatment effects.

For the present RCT, we tested ASET against another potentially active treatment (expressive writing) and also against a control treatment (journaling). The ASET intervention was based on a cognitive intervention previously used for participants with NSSI histories. Specifically, Hooley and St. Germain [18] created a brief intervention that, during one in-lab session, significantly increased positive self-worth and decreased the amount of time participants were willing to endure pain compared to two comparison conditions. ASET was designed to test whether a similar cognitive intervention, administered online, would decrease self-criticism over time and to test whether these decreases would extend to subsequent decreases in NSSI compared to alternative interventions.

We also tested an intervention that involved expressive writing (EW). Expressive writing, a procedure first developed by Pennebaker and Beall [19] involves writing about stressful or upsetting experiences. Many years of research have established that expressive writing produces psychological and physical health benefits [20]. Although NSSI is used to regulate negative emotions [12], to date, no study has examined expressive writing as an intervention for NSSI. Finally, we used a journaling (JNL) condition as a comparison control intervention. This involved writing about daily events without any focus on emotional issues. The JNL intervention was developed to control for daily writing (which was integral to both the ASET and the EW conditions).

We predicted that, compared to the control (JNL) condition, ASET treatment would significantly reduce self-criticism, NSSI, and the desire to engage in NSSI. Depressive symptoms were included as a secondary treatment target because there is high comorbidity between depressive symptoms and NSSI [21]. Meta-analytic evidence further suggests that self-esteem (related to self-criticism) longitudinally predicts depression [22]. Similarly, because NSSI tends to be comorbid with suicidal thoughts and behaviors [3, 4], these were also selected as secondary treatment targets. We hypothesized that ASET would reduce depressive symptoms, suicidal thoughts, and suicidal behaviors compared to JNL. We also predicted that compared to the JNL condition, EW would provide general benefits and reduce feelings of depression. However, because EW does not target self-criticism, we did not predict that participants assigned to this condition would show significant decreases in self-criticism, desire to self-injure and engage in NSSI, or engagement in suicidal thoughts and behaviors.

## Method

### Recruitment and participants

This study was conducted in accordance with the Declaration of Helsinki. All study components were approved by the Institutional Review Board at Harvard University and all participants provided informed consent. Adopting the method used by Franklin and colleagues [10], participants were recruited from online forums related to self-injury and severe psychopathology (e.g., reddit.com/r/depression). Research increasingly supports the use of online methods for valid collection of data and shows that online and in-person studies result in similar outcomes across tasks and populations. Such an approach is particularly useful when studying stigmatized or taboo topics, like self-injury. This is because online study assessment allows for greater participant anonymity and privacy, potentially increasing participant comfort in disclosing stigmatized thoughts, behaviors, and symptoms, including self-injury.

**Fig. 1:**
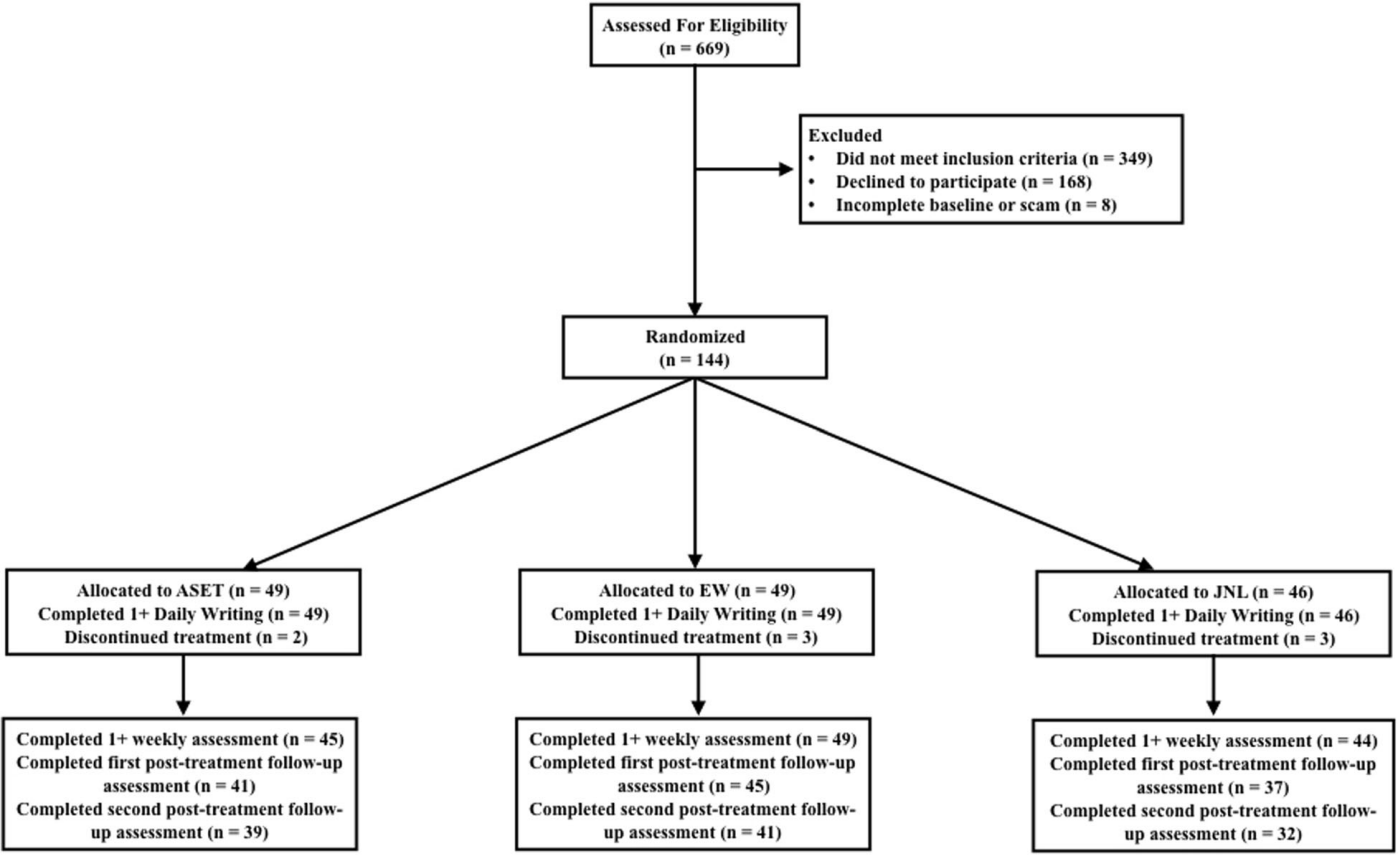
CONSORT diagram illustrating flow through the various stages of the study

After determining eligibility via a screening questionnaire (i.e., 18+ years of age, daily Internet access, English fluency, and 2+ episodes of NSSI in the past month), forum members interested in participating completed an online consent form and an approximately 45-min baseline assessment. Eight participants tried to enter the study multiple times or did not answer the majority (i.e., 90%+) of the baseline assessment questions, indicating problems with validity. These participants were excluded. All participants were entered into the study between July, 2016 and September, 2016. Figure 1 summarizes the flow of recruitment.

The final sample included 144 adults (85.40% female) aged 18–45 years (*M*age = 25.63, *SD* = 5.83) who reported two or more past month NSSI episodes. Most participants lived in the United States (71.53%) and identified as Caucasian (87.50%), with remaining participants identifying as Black (2.08%), Hispanic (2.08%), Asian (3.69%), Native American (3.47%), or Other (3.47%). Additionally, the majority of participants endorsed lifetime (83.33%) and past month psychiatric treatment (52.08%), and many were currently using psychiatric medications (45.83%).

### Treatment conditions

After completion of the baseline assessment, participants were randomly assigned to one of three treatment groups: JNL (*N* = 46), EW (*N* = 49), and ASET (*N* = 49) using randomization software within Qualtrics. Each treatment condition was designed as a brief, daily diary treatment that could be completed from home or from a mobile device anywhere with Internet access. Participants assigned to the ASET condition were asked to write for five minutes each day about something that happened that day that made them feel good about themselves as a person. Participants assigned to the EW condition were asked to write for five minutes each day about something that bothered them or was on their mind that day. Participants in the JNL condition were asked to write for five minutes each day about the events of the day in a general and factually descriptive way (see Table 1 for full directions and Table 2 for examples of writing relevant to each condition). For all conditions, writing responses were monitored daily.

**Table 1:** Daily writing directions

Group	Directions
ASET	Now we’d like you to think about a specific positive characteristic you showed *today*. It doesn’t have to be a big thing -- small things count too! Opening a door open for someone, for instance, is a good example of thoughtfulness. Think back and come up with a specific example. Were you kind, or loyal, or funny? How about being a good friend or a good listener? So long as it is a positive characteristic you showed today, it counts! On the next screen, we would like you to spend around 5 min writing about the positive event you just thought about. As you write try and be as specific as you can and tell us: What happened? How did you feel? Was anyone else involved? If so, how do you think they felt? Are there other examples of times you showed these qualities? The focus of your writing should be on what you did that made you feel good about yourself. All of your writing is COMPLETELY CONFIDENTIAL. No one is judging you. And don’t worry about spelling, sentence structure, or grammar. The only rule is that once you begin writing, you write continuously for about 5 min.
EW	Now we’d like you to think about something that has been on your mind or has concerned or worried you *today*. On the next screen, we would like you to write for around 5 min about a moment from TODAY that’s been on your mind or bothered you in some way. Just use this opportunity to explore your emotions and thoughts about your chosen moment. You might write about: Anything stressful you’re currently experiencing Your relationships with others, including parents, lovers, friends, or relatives Anything concerning your past, your present, or your future Who you have been, who you would like to be, or who you are now The focus of your writing should be on your emotions and thoughts about anything from today. All of your writing is COMPLETELY CONFIDENTIAL. No one is judging you. And don’t worry about spelling, sentence structure, or grammar. The only rule is that once you begin writing, you write continuously for about 5 min.
JNL	Now we’d like you to think about how you spent your time *today*. On the next screen, we would like you to write for 5 min about how you spent your time TODAY. Do NOT include any emotions, feelings or opinions in your writing. Instead, we’d like for you to write in a factual, descriptive way. The focus of your writing should be on the factual aspects of your day’s events and activities. All of your writing is COMPLETELY CONFIDENTIAL. No one is judging you. And don’t worry about spelling, sentence structure, or grammar. The only rule is that once you begin writing, you write continuously for about 5 min.

**Table 2:** Examples of daily writing

ASET	So I was helpful today! Today I was into my apartment and I saw my neighbor struggling to get her luggage up the stairs. She’s a bit older and it looked like she was struggling with the weight, so I asked her if she’d like some help. She said yes, so I carried her luggage all the way up to the 3rd floor. It felt good to help her out because she looked kind of frail, and she was really sweet and grateful. We chatted a bit after about her life, and I liked talking to her. I think she enjoyed talking to me too. I was glad that I was able to make her day a little easier.
EW	Graduation is coming up fast, and I’m really stressed out about what I’m going to do when college is over. I feel as if everyone already has a job lined up, and I’ve still got nothing. And so many of my friends are leaving for graduate school. It’s like everyone has the next stages of their lives figured out, and I’m stuck. Should I move back home with my parents? Or maybe live somewhere cheap and work a couple of minimum wage jobs until something works out? There are so many options, but I don’t actually know which one to take.
JNL	I woke up at 11 am and I took a shower and washed my hair. I got dressed and made my breakfast. I had a bowl of cereal, a banana, and a coffee. Then I got in my car and drove to the office where I work. When I got to work, I answered five emails and went to a meeting. The meeting took until 12:30. I went to a nearby restaurant to buy some lunch. I came back to work and sent more emails. I took a break to talk with my work friends. After work I went to the gym and I ran on the treadmill. Then I drove home. I ordered takeout from a nearby Chinese restaurant and browsed the internet. Then I ate my takeout and called my parents. Then I called my friend to make plans for the weekend.

Participants were asked to complete daily writing assignments as well as brief weekly assessments during the treatment month (i.e., 28 days). Four weeks after the end of treatment participants were contacted again (i.e., 1-month follow up; 8 weeks after baseline; *N* = 123 (85.42%) to complete the first follow-up assessment. A second and final follow up occurred 8 weeks later (i.e., 3-month follow-up; 16 weeks after baseline; *N* = 118 (82.64%).

To maintain participant anonymity, participants were asked to use an email address without identifiable information (e.g., their date of birth or legal name). During the writing phase of the study, participants were emailed daily at 4:00 p.m. (adjusted to their time zone) with a reminder to complete the daily writing assignment. They were also provided with a link to Qualtrics to complete this assignment. Participants were compensated at the end of each week via Amazon, Starbucks, or iTunes gift cards. Participants were compensated $10 for completing the baseline assessment, $2 for each daily writing they completed, $5 for each weekly assessment completed during the treatment month, and $20 for each follow-up assessment completed. To increase participant engagement in the active treatment phase, participants were given a $24 bonus for completing at least 26/28 of the daily writing assignments in addition to each weekly assessment. Additionally, participants who completed at least 20/28 daily writing assignments were entered into a drawing for one of ten $50 gift cards.

The integrity of the treatment was monitored daily. If participants did not submit their daily writing by 2:00 a.m. (personalized to their time zone) or if they submitted a response that did not follow the instructions for their assigned condition (e.g., they included emotional content in the JNL condition), a study coordinator emailed them to remind them to complete the writing assignments daily, to remind them of the compensation structure of the study, and/or to remind them to follow the study guidelines in their writing. Not including missed responses, 0.02%, 5.38%, and 7.04% of daily writings in the EW, JNL, and ASET conditions respectively prompted experimenter emails. These were sent primarily because responses were too short (all three conditions), they did not include the requisite focus on doing something positive or contained too much overall negative content (ASET condition only), or they included emotional content (JNL condition only).

### Measures

#### Demographic information

We assessed basic demographic information including age, sex, gender, and race. Additionally, we assessed lifetime and past month use of psychiatric treatment as well as current use of psychiatric medications.

#### Modified self-injurious thoughts and behaviors interview (SITBI; [23])

The SITBI is typically a semi-structured interview used to assess the presence, frequency, and characteristics of self-injurious thoughts and behaviors, including suicidal and nonsuicidal self-injury. The interview has strong interrater reliability (average κ = .99) and strong convergent and construct validity, indexed by its association with other measures of self-injurious thoughts and behaviors [23]. As with other online research studies [24, 25], we used an online version of the SITBI to assess history of self-injurious thoughts and behaviors. Prior research suggests that online and in-person versions of the SITBI produce similar estimates [26].

In addition to questions assessing lifetime history and frequency of self-injurious thoughts and behaviors, the SITBI was used to asses self-reported desire to discontinue and likelihood of future NSSI from 0 (not at all) to 4 (extremely).

#### Beck depression inventory – II (BDI-II; [27]

The BDI-II is a 21-item self-report questionnaire assessing symptoms of depression. All items are rated on a four point scale. Higher scores on the BDI-II index more severe depressive symptoms. The BDI-II demonstrates high internal consistency [27] and strong convergent and discriminant validity among psychiatric outpatients [28]. Cronbach’s alpha for the BDI-II was excellent, ranging from.92 to.96 at all time points.

#### Self-rating scale (SRS; [16])

The SRS is an 8-item measure that assesses self-critical cognitions (e.g., “I often feel inferior to others,” “I am socially inept and socially undesirable”). Items are answered on a 7-point Likert scale, ranging from strongly disagree to strongly agree, with higher scores indexing higher levels of self-criticism. The SRS demonstrates good internal reliability and can differentiate groups with and without NSSI histories [16, 29]. Cronbach’s alpha for this measure was high and ranged from.84 to.94 at all assessments.

#### Post study assessment

During the final follow-up assessment, participants were questioned about the acceptability of the treatment they received. Specifically, they were asked to respond to the following statements using a scale from 1 (strongly disagree) to 7 (strongly agree): “*I enjoyed writing each day,” “I found the writing task annoying,” “I didn’t understand why I was doing the writing task,” “I plan to keep writing each day because I found it really helpful.”*

### Data analytic plan

#### Retention and missing data

Retention rates were calculated for each week during the treatment month and at the end of the one-month and three-month post-treatment follow ups. There were no significant treatment group differences in retention rates across any of these assessment points. Missing data were minimal (7.1%) and did not differ across treatment groups (*p* > .05).

#### Outcomes

Primary outcomes were NSSI episodes and self-criticism. We also examined the effects of treatment on depression, desire to discontinue NSSI, likelihood of future NSSI, days of active suicide ideation, and days of suicide plans.

#### Statistical models

We utilized mixed-effects regression models for between-group analyses examining the effect of treatment group on outcomes at the end of treatment and post-treatment follow-up assessments. These models allow for inclusion of participants with missing data; therefore, all participants who completed baseline assessments and at least one end-of-treatment or post-treatment assessment were included in analyses.

All mixed effects models included an interaction term between treatment group and assessment time point as fixed effects, and a random intercept for each participant. Additionally, all models controlled for baseline levels of the outcome variable. We fit linear mixed-effects models using the R package lmerTest [30] for continuous outcome measures (self-criticism, depression, desire to discontinue NSSI, likelihood of future NSSI). NSSI episodes, days of active suicide ideation, and days of suicide plans are count variables that tend to be positively skewed and include an excess of zeros. Accordingly, we fit zero-inflated negative binomial mixed-effects models using the R package glmmTMB [31] for these outcome variables.

Pairwise comparisons between treatment groups at all assessment points (end of treatment, post-treatment follow-up assessments one and two) were assessed with the R package lsmeans [32]. To examine overall clinical change (regardless of treatment group) from baseline to the end of treatment, we conducted paired samples *t*-tests for all outcome variables using SPSS.

#### Overview of treatment-related analyses

We were primarily interested in the overall effects of treatment group (ASET vs. EW vs. JNL). Therefore, resembling intention-to-treat tests, our analyses included all participants who completed baseline assessments, were randomized to a treatment group, and completed at least one other assessment at any time point, regardless of whether they completed all daily writings. Although treatment participation differed across treatment groups, as reported below, there were no significant differences in retention rates for assessments between groups. Controlling for the number of completed daily writings also did not change our findings in any important ways. Similarly, with the exception of self-criticism (see footnote 1), controlling for baseline differences in depression did not change results in any way.

#### Treatment and follow-up analyses

We sought to examine whether treatment group significantly predicted self-criticism, depression, and self-injurious thoughts and behavior variables (including NSSI and suicidal thoughts and behaviors) at the end of the treatment month, as well as whether these effects were maintained after treatment ended. Therefore, we used an interaction term to test the effects of treatment group across three time points (end of treatment, 1-month post-treatment follow-up and 3-month post-treatment follow-up). All analyses control for baseline levels of the relevant outcome variable by using baseline scores as a covariate in the model.

#### Suicidal behavior analyses

Suicidal behavior is a low base rate behavior, especially across short time periods. Thus, as with other similar treatment studies [10], we calculated a suicidal behavior variable that included all behaviors throughout the treatment month and at post-treatment follow-up assessments. Because this variable was calculated as the sum of all suicidal behaviors across all time points, we fit a model with treatment group as a single predictor variable, rather than as an interaction term with assessment time point. The zero-inflated portion of the model was also specified with this fixed effect.

## Results

There were no significant demographic or psychiatric treatment history differences among the three groups (all *p*s > .05). Additionally, there were no group differences in self-criticism at baseline (*p* = .34), or in self-reported episodes of past week, month, or year NSSI, suicide ideation, suicide plans, or suicide attempts (all *p*s > .05). However, there were significant differences in baseline depression (*F*(2) = 3.70, *p* = .03). Probing these differences in more detail, Bonferroni corrected post-hoc tests indicated that participants in the ASET condition had significantly lower depression scores than participants in the EW (*p* = .04), but not the JNL condition at baseline; no other group differences were statistically significant. Means for all baseline measures are provided in Table 3.

**Table 3:** Baseline scores for clinical measures

	ASET (*n* = 49) *M* (SD)	EW (*n* = 49) *M* (SD)	JNL (*n* = 46) *M* (SD)
Self-criticism	43.59(8.93)_a_	44.73(7.61)_a_	42.07(9.60)_a_
Depression	33.04(14.53)_a_	40.06(13.65)_b_	39.29(13.30)_b_
Self-cutting episodes	2.33(3.04)_a_	4.02(6.82)_a_	2.89(7.21)_a_
Overall NSSI episodes	7.20(8.30)_a_	10.22(12.26)_a_	9.59(13.47)_a_
Desire to discontinue NSSI	2.29(0.95)_a_	2.12(1.21)_a_	2.27(1.31)_a_
Likelihood of future NSSI	3.06(1.08)_a_	3.15(1.04)_a_	3.00(0.98)_a_
Suicide ideation	9.00(10.53)_a_	10.63(10.89)_a_	12.98(11.49)_a_
Suicide plans	4.96(8.42)_a_	4.02(7.32)_a_	5.57(7.76)_a_
Suicidal behaviors	0.45(1.68)_a_	0.16(0.55)_a_	0.33(0.76)_a_

*Note*. *ASET* Autobiographical Self-Enhancement Training, *EW* Expressive Writing, *JNL* Journaling. Means with different subscripts are significantly different at *p* < .05 from other means within in the same row

### Treatment participation

All participants completed at least one daily writing assignment. Most participants (77.08%) completed more than 21 daily writings with similar proportions across groups (ASET group: 67.35%, EW group: 83.67%, JNL group: 80.43%). However, groups differed significantly in the total number of daily writings completed (*F*(2) = 3.69, *p* = .03). Bonferroni corrected post-hoc tests revealed that participants completed significantly more daily writing assignments in the EW condition (*M* = 23.65; *SD* = 5.18) compared to the ASET condition (*M* = 21.31, *SD* = 7.36; *p* < .05). There were no significant differences in the number of daily writings completed by participants in any other condition.

### Retention rates for follow-up assessments

Retention rates gradually dropped or remained stable from treatment week one to treatment week four (i.e., 95.14%, 92.36%, 86.81%, 87.50% respectively). A total of 95.83% of participants completed at least one of the weekly assessments during the treatment month and 81.25% of participants completed all follow-ups, with no differences in completion rates among groups. During post-treatment months, retention rates remained relatively stable across follow-up assessments at 1-month (85.42%) and 3-months post-treatment (82.64%), again with no differences across treatment groups.

### Overall clinical change

**Fig. 2:**
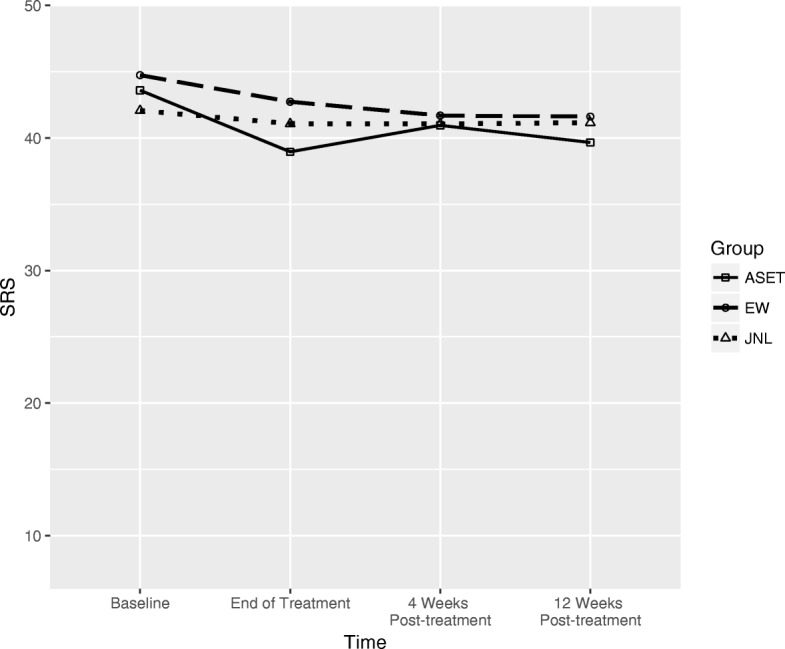
Self-criticism by treatment group. The ASET group reported significantly less self-criticism at the end of treatment than the JNL group (*p* = .047), but not the EW group (*p* = .16). However, this effect was not maintained through either the first (*p* = .46) or second (*p* = .41) posttreatment follow-up assessment

**Fig. 3:**
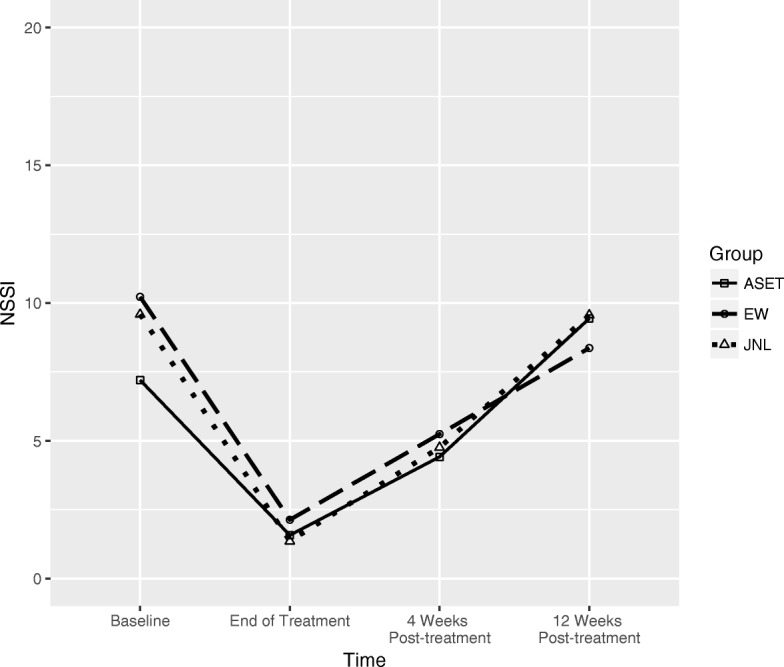
NSSI episodes by treatment group. There was a significant reduction in NSSI from baseline to the end of treatment (*p* = .02), but no significant effects of treatment group at the end of treatment or at post-treatment follow-ups

Regardless of treatment group, participants demonstrated significant reductions in self-criticism (*t*(125) = 3.69, *p* < .001) and overall NSSI episodes (*t*(125) = 2.38, *p* = .02) from baseline to the end of treatment. These findings are illustrated in Figs. 2 and 3. There were also significant decreases in depression (*t*(122) = 7.91, *p* < .001) and suicide ideation (*t*(121) = 2.41, *p* = .02) over this same period. Desire to discontinue NSSI, likelihood of future NSSI, or suicide plans, or suicidal behaviors remained unchanged.

### Treatment analyses

#### Self-criticism^1^

The ASET group reported significantly less self-criticism at the end of treatment than the JNL group (*B* = − 4.31, *SE* = 1.81, *p* = .047), but not the EW group (*p* = .16). However, this effect was not maintained through either the first (*p* = .46) or second (*p* = .41) post-treatment follow-up assessment.

#### Depression

No significant effects of treatment group were detected for depression at the end of treatment or post-treatment follow-up assessments.

#### Overall NSSI episodes

No significant effects of treatment group were detected for overall NSSI episodes at the end of treatment or at the 1- or 3-month follow-up assessments.

#### Desire to discontinue NSSI

No significant effects of treatment group were detected for desire to discontinue NSSI at the end of treatment or at the follow-up assessments.

#### Likelihood of future NSSI

No significant effects of treatment group were detected for likelihood of future NSSI at the end of treatment or at the follow-up assessments.

#### Suicide ideation

The JNL group reported significantly fewer days of suicide ideation than the EW group (but not the ASET group) at the end of treatment (*B* = − 0.62, *SE* = 0.26, *p* = .04); this effect did not reach significance at the 1-month post-treatment follow-up assessment (*p* = .33), but was maintained at the 3-month follow-up assessment (*B* = − 0.57, *SE* = 0.19, *p* = .001). There was also a trend toward the ASET group reporting significantly fewer days of suicide ideation at the end of treatment compared with the EW group (*B* = − 0.60, *SE* = 0.27, *p* = .07). This did not reach significance at the 1-month post-treatment follow-up assessment (*p* = .52), but was apparent at the 3-month post-treatment follow-up assessment (*B* = − 0.50, *SE* = 0.21, *p* = .048).

#### Suicide plans

No significant effects of treatment group were detected for suicide plans at the end of treatment or post-treatment follow-up assessments.

#### Suicidal behaviors

No significant effects of treatment group were detected for suicidal behaviors throughout the treatment month or post-treatment follow-up assessments.

#### Acceptability of treatment

There were no significant group differences in self-reported understanding of the reason for completing daily writing assignments, or for self-reported helpfulness of the daily writing assignment (*p*s = .10 and.64 respectively). However, there were significant group differences in finding the writing task enjoyable (*F(*2) = 5.55*, p =* .01) and finding the writing task annoying (*F*(2) = 3.10, *p = .*05). Bonferroni corrected post-hoc tests indicated that participants in the ASET condition found the daily writing task to be significantly less enjoyable than participants than the EW condition (*p* = .01) but not the JNL condition, though effects were trending toward significance (*p* = .07). Similarly, Bonferroni corrected post-hoc tests indicated that participants in the ASET found the writing task was significantly more annoying than participants in the EW condition did (*p* = .047), with no other significant group differences.

## Discussion

This study examined the efficacy of ASET, a novel cognitive intervention, in reducing self-criticism, as well as NSSI and suicidal thoughts and behaviors, among adults with a recent history of NSSI. Supporting our hypotheses, the ASET intervention produced greater reductions in self-criticism at the end of treatment compared to the control JNL condition. There was also a trend toward participants in the ASET condition showing lower levels of suicide ideation at post-treatment compared to participants assigned to EW. Reductions in self-criticism did not persist past the active treatment period, but the effect for suicide ideation was significant at the 3-month post-treatment follow-up. These results are especially notable in light of fewer completed daily writings and participant reports that ASET was less enjoyable than the other two conditions. Although participants liked the EW intervention overall, it was no more efficacious than ASET or JNL for any treatment outcome.

Replicating and extending previous findings [18], the current results demonstrate that an intervention targeting self-criticism decreases both self-criticism and suicide ideation among individuals with NSSI. It also warrants mention that, overall, all of our writing-based approaches provided clinical benefits and reduced episodes of NSSI. Given the high prevalence of NSSI behaviors in clinical and nonclinical populations [2, 33] as well as their concerning associations with suicide [5], highly scalable and disseminable treatments are needed to reduce these dangerous behaviors. These findings provide further evidence supporting the feasibility and acceptability of online interventions for NSSI.

Although these results are promising, they should be interpreted with caution. Despite significant reductions in self-criticism following the ASET intervention, levels of self-criticism continued to remain quite high for individuals in all treatment groups and reductions did not persist after treatment termination. Moreover, many clinical outcomes remained unchanged, such that no treatment was significantly more successful than the others in reducing overall NSSI episodes, suicide plans, suicidal behaviors, depression, desire to discontinue NSSI, or likelihood of engaging in future NSSI.

The finding that the JNL treatment reduced suicide ideation compared to EW was unexpected and warrants consideration. It is also interesting in light of recent findings from another RCT. Celano and colleagues [34] compared a positive psychology-based intervention with a cognition-focused control intervention among high-risk patients discharged from inpatient care. The cognition-focused intervention, which had some similarities to our JNL intervention, required participants to recount three events occurring each day, in an emotionally neutral manner, for the duration of treatment. Contrary to expectation, the cognition-focused intervention resulted in significantly larger reductions in hopelessness at the 6-week follow-up, and significant reductions in depression and suicidality at both 6 and 12 week follow-up assessments. Results suggest that encouraging people who may be at high risk for self-injurious thoughts and behaviors to think about their days in the absence of emotional content could be palliative. However, it is important to keep in mind that the JNL condition did not outperform EW or ASET in reducing NSSI episodes or other self-injury outcomes. Additional studies are needed to more fully evaluate the potential benefits of writing about the events of one’s day in a non-emotional manner.

Findings for the current study should also be considered in the context of the study’s limitations. First, participants were predominantly white and female young adults who were recruited from online forums related to self-injury and psychopathology. It is therefore unclear whether these findings would generalize to individuals of other ages, ethnicities, or genders recruited in other ways. All outcome data were also based on self-report. This is not unusual for studies of this type, as participant anonymity helps decrease the potential for demand characteristics. Nonetheless we acknowledge this as a limitation. The fact that participants were paid for their participation may also have impacted their willingness and motivation to complete the daily writing assignments and our moderate sample sizes may have precluded our ability to detect all but the largest treatment effects on clinical outcomes. Finally, all participants completed standard, once-daily, five-minute writing assignments. It is possible that higher dosages of each treatment (e.g., greater number of writing assignments per day; longer length of writing assignments) would produce greater reductions in self-criticism and self-injurious thoughts and behaviors.

One unexpected finding from the current study was that all participants improved over the course of treatment, regardless of the treatment condition to which they were assigned. In other words, all forms of daily writing were helpful. More specifically, all approaches reduced episodes of NSSI, self-criticism, depression, and suicide ideation. This could reflect regression to the mean, although improvement was not noted across all outcome variables. Another non-specific factor that should be considered is participants’ knowledge that members of the research team were paying attention to them and reading their daily writings. This sense of connection, however temporary, may have been helpful. Future studies involving journaling would do well to consider the possibility that this may be a more active treatment approach than might be apparent at first glance. With hindsight, it would have been advantageous to also include another comparison group that received no online intervention whatsoever, or one in which participants were receiving equivalent contact with researchers. This would have allowed us to determine more about whether the simple act of journaling in an emotionally neutral manner is beneficial for individuals with NSSI.

Overall, this study contributes important new information about the feasibility and efficacy of novel online interventions for NSSI. Findings further suggest that although self-criticism may be an important treatment target in NSSI, altering self-criticism appears to be particularly difficult for individuals with severe and recent NSSI histories and high levels of depression. People who engage in NSSI do not appear to like focusing on their positive attributes. This may be because their negative beliefs about the self are well established and entrenched. It is possible that more direct (one-on one) approaches are needed to provide additional direction and support for overcoming the cognitive challenges presented by considering alternative and more positive aspects of their identities. Alternatively, more covert or implicit approaches capable of enhancing positive self-concept without triggering resistance could be considered. In contrast, writing about negative emotions, such as occurred in the EW condition, appears to be much more enjoyable for people who engage in NSSI. One reassuring aspect of our data is the finding that such a focus on negative emotions in the context of expressive writing does not increase the likelihood of negative outcomes. This seems important to know.

## Conclusion

Future research should examine the effects of ASET on self-criticism and self-injurious thoughts and behaviors among individuals with less severe clinical presentations. The potential for online interventions such as the one described here to prevent the development of NSSI should also be considered. Adolescence is a time of increased self-criticism and increased risk for NSSI. Interventions that focus attention on positive self-attributes may provide some protection against the development of NSSI. Further research is also needed to test the benefits of ASET as an adjunct to existing treatments, in addition to a stand-alone intervention. Although much remains to be learned, the current findings demonstrate that ASET is a feasible and potentially promising intervention that is cost effective, easily accessible, and highly scalable. The benefits of non-emotional journaling also warrant increased consideration.

## Abbreviations

ASET: Autobiographic Self-Enhancement Training
EW: Expressive Writing
JNL: Journaling
NSSI: Nonsuicidal self-injury

## Footnotes

1 When controlling for baseline depression, the stronger effect of ASET than JNL in reducing self-criticism at the end of treatment was no longer significant (*p* = .11); however, ASET and JNL did not significantly differ in depression at baseline, and depression did not have a significant main effect (*p* = .22).
